# Public health application of predictive modeling: an example from farm vehicle crashes

**DOI:** 10.1186/s40621-019-0208-9

**Published:** 2019-06-17

**Authors:** Shabbar I. Ranapurwala, Joseph E. Cavanaugh, Tracy Young, Hongqian Wu, Corinne Peek-Asa, Marizen R. Ramirez

**Affiliations:** 1 Injury Prevention Research and Department of Epidemiology, University of North Carolina at Chapel Hill, 137 E Franklin St, Suite 500, CB# 7505, Chapel Hill, NC 27599 USA; 2 Department of Epidemiology, College of Public Health, University of Iowa, Iowa City, IA USA; 3 Injury Prevention Research Center, Department of Occupational and Environmental Health, College of Public Health, University of Iowa, Iowa City, IA USA; 4 Department of Biostatistics, University of Iowa, Iowa City, IA USA; 5 Division of Environmental Health Sciences, University of Minnesota, Minneapolis, MN USA

**Keywords:** Forecasting, Predictions, Decision support techniques, Motor vehicles

## Abstract

**Background:**

The goal of predictive modelling is to identify the likelihood of future events, such as the predictive modelling used in climate science to forecast weather patterns and significant weather occurrences. In public health, increasingly sophisticated predictive models are used to predict health events in patients and to screen high risk individuals, such as for cardiovascular disease and breast cancer. Although causal modelling is frequently used in epidemiology to identify risk factors, predictive modelling provides highly useful information for individual risk prediction and for informing courses of treatment. Such predictive knowledge is often of great utility to physicians, counsellors, health education specialists, policymakers or other professionals, who may then advice course correction or interventions to prevent adverse health outcomes from occurring. In this manuscript, we use an example dataset that documents farm vehicle crashes and conventional statistical methods to forecast the risk of an injury or death in a farm vehicle crash for a specific individual or a scenario.

**Results:**

Using data from 7094 farm crashes that occurred between 2005 and 2010 in nine mid-western states, we demonstrate and discuss predictive model fitting approaches, model validation techniques using external datasets, and the calculation and interpretation of predicted probabilities. We then developed two automated risk prediction tools using readily available software packages. We discuss best practices and common limitations associated with predictive models built from observational datasets.

**Conclusions:**

Predictive analysis offers tools that could aid the decision making of policymakers, physicians, and environmental health practitioners to improve public health.

## Introduction

Predictive models or algorithms are routinely utilized to predict many everyday events or decisions. The list of such outcomes include daily or inclement weather events (Saha et al. 2014); department store purchases that companies use to provide coupons at grocery stores (Wu and Bryniolfsson 2013; Schoen et al. 2013); or targeted advertisement and news stories that we receive on Facebook, Yahoo, travel websites, or elsewhere. The information needed for such prediction may, for example, come from an individual’s browsing history or grocery shopping habits. Many variables are needed to predict a future event with good accuracy, with an implicit assumption that the predicted event has consistent explanatory factors. For example, to predict tomorrow’s weather in a given city, we will need information on today’s weather in that city, today’s weather in all places surrounding that city, the wind speed, and the season and annual averages based on past weather trends. In the current highly digitized world with Big Data, large sets of variables with sophisticated predictive algorithms may allow forecasting a future event with increasing fidelity.

In healthcare settings, medical tests, (e.g., blood pressure or cholesterol measurements) are one form of predictive data that allow physicians to assess the probability that the patient may (or may not) have an adverse event in the (near or distant) future. We have utilized many such medical tests to predict good or bad health outcomes, for example, to predict breast cancer (Gail et al. 2007; Quante et al. 2012) and 5-year or 10-year risks of cardiovascular diseases (Keys et al. 1972; Kleinbaum et al. 1971; Wilson et al. 1998). The latter work (Keys et al. 1972; Kleinbaum et al. 1971; Wilson et al. 1998) led to the development of a web-based tool to forecast the 10-year risk of cardiovascular disease, predicted by age, sex, smoking status, blood pressure (including medications), and cholesterol levels (AHA 2017). Similarly, predictive algorithms are also now being used to estimate the probability that an individual may suffer from an opioid overdose in a defined future period (Glanz et al. 2018). Such predictive algorithms are helpful for a physician, counselor, health education specialist, social worker, police or parole officer, policy maker or other professionals to identify individuals at an increased risk of having certain adverse outcome within a given timeframe. Predictive models can also be used to help educate target populations so that individuals can understand their personal risks given their unique set of demographics, behavioral characteristics or environmental / social conditions.

Notably, predictive models serve a different purpose than causal (or associational) models and their resultant measures like relative (ratio) measures of risks, rates, odds or hazards. In multivariable modeling, causal or associational measures (risk ratios, rate ratios, odds ratios or hazard ratios) examine the partial relationship (or association) between a single independent variable with a health outcome (dependent variable) on a ‘population level.’ Causal measures tell us for example the effectiveness of population level interventions in improving public health or the population level impact of harmful exposures and their resulting public health burden. Thus, causal models help in population-level decision making, especially in formulating policy or even treatment decisions that affect large populations (e.g., firearm safety laws, laws mandating use of prescription monitoring programs or standardized treatment regimens for treating cancers or HIV-AIDS). However, causal measures do not quantify the risk of the adverse event for a particular individual with a particular set of variable values. Predictive models offer a complementary means to aid decision making at the individual or situational level, which causal (or associational) models are unable to address. For example, while comparing a sample of individuals who were exposed to a certain condition to another sample of individuals who were not exposed to that condition, we may discover that the risk of a hypothetical adverse event may be twice as high among the exposed than the unexposed, i.e., a risk ratio of 2. While on a population or sample level this may hold true, the risk of actually experiencing the adverse event for each individual in the exposed and unexposed subsets differ. Indeed, some individuals in the exposed subset will never experience the adverse event, and some individuals will experience the adverse event even when they were not exposed. A predictive algorithm allows us to identify or measure subject-specific probabilities of experiencing an adverse events, thereby aiding decision making at an individual or situational level.

Predictive models can be developed using the same traditional regression analyses methods used for causal modelling (e.g. linear, log binomial, logistic, Poisson, Cox regression). In this manuscript, we illustrate the development of a simplistic predictive algorithm for an injury-related outcome that incorporates our prior understanding of the substantive area and utilizes a large database to predict the probability of an adverse event.

Predictive models have widespread applicability in the area of injury and violence prevention, especially as the field continues to develop and implement interventions that target individual behaviors. For example, a physician could use predictive tools based on a patient’s diagnosis, mental and physical health history and previous medication prescriptions to titrate the supply of opioid pain medication for that patient so that the pain is adequately controlled while also reducing the risk of an opioid use disorder. Using similar patient-history driven predictive tools, a mental health counselor could advise reduced access to a firearm for someone at high risk of committing suicide. A particular focus of our research is motor vehicle crashes, a leading cause of death in rural agricultural communities. Predictive models can aid prevention efforts that target individual protective behaviors while driving or operating agricultural equipment on roadways. Examples of protective behaviors include use of seat belts, installation of rollover protective devices in tractors and safe driving behaviors (following traffic regulations).

We used farm vehicle equipment involved motor vehicle crash (henceforth, farm crash) data from nine Midwestern states in the United States from 2005 to 2010 to build models for forecasting the risk of injury or death in a farm crash. For this case example, we employ the logistic regression framework, as this regression technique best addresses the nature of the data. However, the methods described herein can be easily replicated in other datasets where outcome distributions favor the use of alternative modeling frameworks.

We demonstrate model building approaches for predictive models using prior knowledge of the substantive area. We further demonstrate model fitting approaches, model validation techniques using external datasets, and the calculation and interpretation of predicted probabilities. Additionally, we share two risk prediction tools that can be used by lay and scientific audiences to predict their own or hypothetical risks of being injured should they be involved in a farm crash. Lastly, we discuss best practices and common limitations associated with predictive models.

## Methods

### Example data

Transportation is the leading cause of agricultural-related death, and crashes with farm equipment on roadways present a burden for all roadway users (BLS 2013a, b). Predictive algorithms in transportation research could allow us to identify high crash risk scenarios and provide opportunities to intervene. We used secondary crash data from 2005 to 2010 to assess the risk of injury or death in farm vehicle-related crashes (referred hereon after as crash/ farm crash, unless otherwise mentioned), given crash-level, vehicle-level, and individual-level factors. The data were collected from the departments of transportation (DOT) of Illinois (IL), Iowa (IA), Kansas (KS), Minnesota (MN), Missouri (MO), Nebraska (NE), North Dakota (ND), South Dakota (SD), and Wisconsin (WI). The data include all police-reported crashes, including death, injury, or property damage of at least $500 to $1500, depending on the state. Methods to identify and code farm vehicle crashes from these data have been discussed previously (Harland et al. 2014; Ranapurwala et al. 2016). Multilevel crash data include the characteristics of crashes, involved vehicles, and their occupants. The crash characteristic variables were year, month, and date of the crash, day of the week, time of the day, the state in which crash occurred, season, number of fatalities, number of injured occupants, number of vehicles involved in the crash, manner of collision, ambient lighting at the time of crash, and weather conditions. The vehicle characteristics included vehicle type, vehicle action at the time of the crash, number of occupants, and driver contributing circumstances. The person-level characteristics included age, sex, injury status (no injury or fatal and non-fatal injury), occupant protection information, occupant seating, and driver or passenger status.

Injury severity, coded by the reporting law enforcement officer, was available at the individual-level, defined as no injury, possible injury, non-incapacitating injury, incapacitating injury, or fatality. We developed a new binary injury variable for each individual, such that ‘any injury’ corresponds to non-incapacitating, incapacitating, or fatal injury; and ‘no injury’ corresponds to no or possible injury.

Discrete covariate categories were collapsed into broader but meaningful categories based on a-priori knowledge (Hughes and Rodgman 2000; Peek-Asa et al. 2007; Pinzke and Lundqvist 2004; Costello et al. 2003; Gerberich et al. 1996; Jaarsma and De Vries 2014; Marlenga et al. 2006; Gkritza et al. 2010; Maio et al. 1992; Stephan and Newstead 2014; Russo et al. 2014). Vehicle type was classified as farm vehicle or non-farm vehicle. Number of vehicle occupants was categorized as single or multiple occupant. The crash type was defined as either a single vehicle crash (farm vehicle only), or a multiple vehicle crash (two or more vehicles, one of them being a farm vehicle).

### Statistical analysis

Predictive models were fit using multivariable logistic regression for individual-level data. To accommodate clustering at the crash level, generalized estimating equations (GEEs) with an exchangeable working correlation structure were employed to fit the models. Three types of models were formulated. First, we added a set of non-modifiable variables, which cannot be practically intervened on, to determine changes in injury probability given the non-modifiable factors (model 1). The non-modifiable variables included state, season, weather, time-of-crash, number of involved vehicles, equipment type, and age and sex of the occupant. Next, we added semi-modifiable factors that can be indirectly intervened upon or are consequences of the modifiable factors (model 2). These included ambient light, manner of collision, vehicle action, number of occupants in the vehicle, and the occupant type. Lastly, we added modifiable factors, which may be directly intervened on, to the model (model 3). These included driver contributing circumstances and occupant protection. Non-modifiable, semi-modifiable, and modifiable factors were classified a-priori based on previous literature (Hughes and Rodgman 2000; Peek-Asa et al. 2007; Pinzke and Lundqvist 2004; Costello et al. 2003; Gerberich et al. 1996; Jaarsma and De Vries 2014; Marlenga et al. 2006; Gkritza et al. 2010; Maio et al. 1992; Stephan and Newstead 2014; Russo et al. 2014), expert knowledge, and consensus among the research team members.

We did not include variables that were highly collinear with other variables that better explained the outcomes. Seating was highly correlated with the driver status (driver/ passenger), and month was highly correlated with season; consequently, these two variables were removed from the predictive model. Day of the week was removed because it did not predict injury. Occupant age in 10-year age categories provided better penalized model fit than continuous or log-transformed age. Multiple imputation (five imputed datasets), using the fully conditional specification methods for logistic regression, was performed to impute missing values for vehicular action (5.9%), driver circumstances (14.8%), and occupant protection (21.4%) with no interactions. All models were fit to each of the five imputed datasets. The resulting regression coefficients from the imputed datasets were then pooled to obtain the final coefficients that are reported here.

### Internal validation

Model fit was assessed in three ways. First, by using the quasi-likelihood information criterion (QIC), a variant of AIC designed for models fit using GEEs (Pan 2001). Smaller values of QIC (or AIC or BIC) correspond to models that provide better penalized fit (Akaike 1973; Schwarz 1978). Second, by calculating the concordance statistic (or c-statistic), which estimates the area under the receiver operating characteristic curve (AUC). The c-statistic ranges from 0.50 to 1, with higher values corresponding to models that provide better discrimination between outcome occurrence and non-occurrence (Gail et al. 2007; Quante et al. 2012). Lastly, model fit was assessed by estimating an expected outcome count using the model coefficients in the same dataset from which the coefficients were obtained (Keys et al. 1972). A chi-squared statistic was calculated to compare the expected and observed outcome count to assess statistical difference between the two. Note that all three of these methods are of limited utility because they only evaluate the fitted model based on the same dataset used in the model construction (i.e., the “training data”), and do not examine if the predictive models could accurately predict outcomes in new data (i.e., in “validation data”). Hence validation of the predictive model in external data is also needed.

### External validation

We partitioned 2005–2010 data in three ways to obtain a training dataset and a validation dataset: 1) 2005–2007 data were separated as training data and 2008–2010 as validation data, 2) 2005–2008 data were separated as training data and 2009–2010 as validation data, and 3) 2005–2009 data were separated as training data and 2010 as validation data. We fit models 1–3 using each of the three training datasets and applied the estimated model coefficients to obtain expected outcome counts in the corresponding validation datasets. This allowed us to compare the observed injury/death counts from the validation dataset to the expected injury/death counts from the same validation dataset using predictions based on models 1–3 from the training datasets.

### Predicted probabilities

Predicted probabilities for different regression models can be readily obtained from standard statistical analyses programs such as SAS, STATA, R, etc. For this example, based on the estimated model coefficients from the final fitted logistic regression model (model 3), we constructed some crash scenarios as examples to demonstrate the calculation and interpretation of predicted probability of injury. In our application, the predicted probability (or risk) of injury or death in a farm crash scenario was calculated for each occupant involved in the crash as:

$$ \mathrm{P}\left(\mathrm{Injury}\ \mathrm{or}\ \mathrm{Death}\right)=\frac{\mathrm{Odds}}{1+\mathrm{Odds}}=\frac{1}{1+\left(\exp \left[-\left(\alpha +{\upbeta}_1{C}_1+{\beta}_2{C}_2+\cdots +{\beta}_i{C}_i\right)\right]\right)} $$

Here, P is the predicted probability or risk of injury or death, *C*_1_, *C*_2_, … , *C*_*i*_ are the covariates in the logistic regression model, and *β*_1_, *β*_2_, … , *β*_*i*_ are the regression coefficients for those covariates (Keys et al. 1972; Kleinbaum et al. 1971).

All analyses were conducted using SAS 9.4 (SAS Institute, Cary, NC). Using the model coefficients, we built two interactive risk prediction tools, one using Microsoft Excel (Microsoft, Inc), and one using R-Shiny (RStudio). The study was considered non-human subjects by the Institutional Review Board at the University of Iowa due to the de-identified nature of the secondary data.

## Results

From 2005 to 2010 there were 7094 farm crashes in the nine states, of which 86% (*n* = 6119) were multiple vehicle crashes and 14% (*n* = 975) were single vehicle crashes. Of the 7094 crashes, 10 crashes had missing injury or fatality information, and were subsequently excluded from the analyses. A total of 12,936 vehicles were involved in the 7084 crashes, of which 11,961 vehicles were involved in multiple vehicle farm crashes. There were 14,834 occupants involved in the 7084 crashes, and of these, 2087 (14.1%) had been injured or killed in the crash. The distribution of the crashes, injuries and deaths by state and year are presented in Table 1.

**Table 1:** Distribution of farm vehicle crashes and resulting injuries and deaths by calendar year and state: 2005–2010

	Farm vehicle crashes *N* (%)	Injuries and deaths *N* (rate per 100 crashes)	Deaths *N* (rate per 100 crashes)
Total	7084	2087 (29.5)	163 (2.3)
Calendar Year			
2005	1166 (16.5)	338 (29.0)	21 (1.8)
2006	1114 (15.7)	318 (28.5)	29 (2.6)
2007	1198 (16.9)	336 (28.0)	26 (2.2)
2008	1160 (16.4)	316 (27.2)	22 (1.9)
2009	1196 (16.9)	407 (34.0)	37 (3.1)
2010	1250 (17.6)	372 (29.8)	28 (2.2)
State			
Iowa	1178 (16.6)	421 (35.7)	35 (3.0)
Illinois	1214 (17.1)	421 (34.7)	27 (2.2)
Kansas	700 (9.9)	186 (26.6)	19 (2.7)
Minnesota	850 (12.0)	199 (23.4)	22 (2.6)
Missouri	1084 (15.3)	207 (19.1)	12 (1.1)
North Dakota	253 (3.6)	51 (20.2)	12 (4.7)
Nebraska	536 (7.6)	189 (35.3)	10 (1.9)
South Dakota	232 (3.3)	74 (31.9)	8 (3.4)
Wisconsin	1037 (14.6)	339 (32.7)	18 (1.7)

Table 2 presents regression coefficients from three fitted models: model 1 included non-modifiable factors, model 2 added the semi-modifiable factors, and model 3 included two additional modifiable factors. Comparing the QICs and c-statistics (estimated AUCs) from the three nested models (models 1, 2 and 3), we see that model 3 was the best fitting and was hence selected as the final model.

**Table 2:** Injury / death status, and estimated regression coefficients for non-modifiable, non + semi-modifiable, and non + semi+modifiable risk factors to predict the risk of injury or death in a farm crash: 2005–2010

Variables	Categories	Injured or died		Model coefficients (std. error)		
		Yes	No	Model 1^a^	Model 2^b^	Model 3^c^
Intercept	Intercept (constant)	2087	12,747	−1.75 (0.18)	−1.73 (0.24)	−0.97 (0.25)
Non-modifiable factors						
State	Iowa	421	1858	0.39 (0.10)	0.46 (0.10)	0.42 (0.10)
	Illinois	421	2230	0.19 (0.10)	0.20 (0.11)	0.33 (0.11)
	Kansas	186	1291	−0.19 (0.12)	−0.21 (0.12)	− 0.26 (0.13)
	Minnesota	199	1688	−0.30 (0.11)	−0.24 (0.12)	− 0.08 (0.12)
	Missouri	207	1686	−0.09 (0.11)	−0.21 (0.12)	− 0.34 (0.12)
	North Dakota	51	431	−0.43 (0.21)	−0.50 (0.21)	− 0.56 (0.21)
	Nebraska	189	970	0.16 (0.12)	0.11 (0.12)	0.08 (0.12)
	South Dakota	74	379	0.16 (0.17)	0.14 (0.18)	−0.06 (0.19)
	Wisconsin (referent)	339	2214	0	0	0
Season	Winter (Jan-Mar) (referent)	192	1510	0	0	0
	Planting (Apr-May)	341	2307	0.06 (0.12)	0.20 (0.12)	0.17 (0.12)
	Growing (Jun-Aug)	635	3492	0.30 (0.11)	0.42 (0.11)	0.38 (0.11)
	Harvesting (Sep-Dec)	919	5438	0.19 (0.10)	0.16 (0.10)	0.15 (0.10)
Weather at the time of crash	Clear (referent)	1618	9802	0	0	0
	Cloudy	329	2081	−0.01 (0.08)	− 0.09 (0.08)	− 0.11 (0.08)
	Rain	78	376	0.17 (0.15)	−0.03 (0.16)	0.03 (0.16)
	Snow/sleet/hail/freezing rain/drizzle	22	289	−0.74 (0.28)	−0.89 (0.28)	− 0.87 (0.28)
	Fog/smog/smoke/other	40	199	0.15 (0.22)	0.01 (0.22)	−0.08 (0.23)
Time of crash	12:00–5:59 am (referent)	131	739	0	0	0
	6:00–11:59 am	501	3478	−0.11 (0.13)	0.13 (0.14)	0.17 (0.14)
	12:00–5:59 pm	885	6218	−0.14 (0.13)	0.08 (0.13)	0.08 (0.14)
	6:00–11:59 pm	570	2312	0.41 (0.14)	0.12 (0.14)	0.11 (0.14)
Number of vehicles	Single vehicle	238	827	1.37 (0.10)	1.22 (0.13)	1.22 (0.14)
	Two or more vehicles (referent)	1849	11,920	0	0	0
Equipment type	Farm vehicle/equipment	672	6875	−1.14 (0.06)	−1.02 (0.06)	−1.63 (0.08)
	Non-farm vehicle (referent)	1415	5872	0	0	0
Age	< 16 years age	159	799	0.24 (0.11)	0.04 (0.13)	0.13 (0.14)
	16–24 years age	388	2120	0.24 (0.09)	0.21 (0.09)	0.22 (0.10)
	25–34 years age (referent)	234	1839	0	0	0
	35–44 years age	288	1851	0.15 (0.10)	0.15 (0.10)	0.20 (0.10)
	45–54 years age	325	2326	0.11 (0.09)	0.10 (0.10)	0.13 (0.10)
	55–64 years age	278	1794	0.22 (0.10)	0.24 (0.10)	0.30 (0.10)
	65+ years age	415	2018	0.49 (0.09)	0.51 (0.09)	0.59 (0.10)
Sex	Female (referent)	626	2663	0	0	0
	Male	1461	10,084	−0.19 (0.06)	−0.14 (0.06)	−0.26 (0.06)
Semi-modifiable factors						
Light	Daylight (referent)	1407	10,195		0	0
	Dark-street lights on	27	223		−0.20 (0.25)	−0.27 (0.26)
	Dark-no street lights	553	1882		0.57 (0.10)	0.59 (0.10)
	Other	100	447		0.50 (0.14)	0.42 (0.15)
Manner of collision	Non collision (referent)	294	1301		0	0
	Head-on	127	415		0.40 (0.17)	0.40 (0.18)
	Rear-end	716	2747		0.28 (0.13)	0.28 (0.13)
	Angle, oncoming left turn	361	2343		0.04 (0.14)	0.10 (0.14)
	Sideswipe, same direction	192	2698		−0.85 (0.15)	−0.73 (0.16)
	Sideswipe, opposite direction	152	1462		−0.65 (0.16)	−0.55 (0.16)
	Other	245	1781		−0.21 (0.14)	−0.21 (0.14)
Vehicle action	Heading straight (referent)	1510	6934		0	0
	Turning	133	2489		−0.55 (0.11)	−0.62 (0.11)
	Overtaking/ passing/ changing lanes	280	1750		−0.25 (0.09)	−0.34 (0.10)
	Slowing/stopping	43	608		−0.89 (0.17)	−0.84 (0.18)
	Other	121	966		−0.30 (0.11)	−0.35 (0.11)
Multiple passengers	No (referent)	1392	9908		0	0
	Yes	695	2839		0.28 (0.08)	0.34 (0.08)
Driver	No (referent)	433	1710		0	0
	Yes	1654	11,037		−0.16 (0.09)	−0.08 (0.09)
Modifiable factors						
Driver contributing circumstance	No contributing action (referent)	871	6508			0
	Disregarded traffic regulation	302	1487			0.35 (0.09)
	Reckless, careless, negligent, aggressive driving	407	2510			0.16 (0.08)
	Inattentive/distracted driver	256	1093			0.25 (0.09)
	Other contributing action	251	1149			0.25 (0.10)
Occupant Protection	None (referent)	870	4534			0
	Seat belt	1052	7439			−1.19 (0.09)
	Child safety restraint	59	384			−1.25 (0.20)
	Other restraint/ protection	106	390			0.18 (0.16)
Quasi-likelihood Information Criterion (QIC)				10,844.4	10,077.4	9676.8
AUC (95% CI)				0.69 (0.68, 0.71)	0.75 (0.74, 0.76)	0.78 (0.76, 0.79)

*Abbreviations*: *AUC* Area under the receiver operating characteristic (ROC) curve

^a^model 1 includes non-modifiable factors

^b^model 2 includes non-modifiable and semi-modifiable factors

^c^model 3 includes non-modifiable, semi-modifiable, and modifiable factors

The comparisons of state-by-state expected injuries/ deaths to the observed injury/ deaths from also implies that model 3 best characterized the observed data as compared to models 1 and 2 (Table 3), thereby suggesting that model 3 best estimated the overall number of injuries and deaths.

**Table 3:** Comparison of the expected (model-based) to observed number of injuries in the nine states from 2005 to 2010

State	Total occupants (*N*)	Observed injuries or deaths (*N*)	Non-modifiable (Model 1) QIC = 10,844.4 AUC = 0.69 (0.68, 0.71)		Non + semi-modifiable (Model 2) QIC = 10,077.4 AUC = 0.75 (0.74, 0.76)		Non + semi + modifiable (Model 3) QIC = 9676.8 AUC = 0.78 (0.76, 0.79)	
			Avg. pred. Prob.	Expected injuries/ deaths (*N*)	Avg. pred. Prob.	Expected injuries/ deaths (*N*)	Avg. pred. Prob.	Expected injuries/ deaths (*N*)
IA	2279	421	0.1804	411	0.1820	415	0.1818	414
IL	2651	421	0.1600	424	0.1602	425	0.1594	422
KS	1477	186	0.1251	185	0.1246	184	0.1242	183
MN	1887	199	0.1008	190	0.1044	197	0.1017	192
MO	1893	207	0.1135	215	0.1169	221	0.1197	227
ND	482	51	0.1052	51	0.1066	51	0.1064	51
NE	1159	189	0.1634	189	0.1641	190	0.1619	188
SD	453	74	0.1549	70	0.1586	72	0.1592	72
WI	2553	339	0.1383	353	0.1367	349	0.1365	348
Total	14,834	2087	0.1408	2089	0.1419	2104	0.1415	2099

*Abbreviations*: *Avg. pred. Prob.* Average predicted probability, *AUC* Area under the receiver operating curve, *QIC* Quasi-likelihood information criteria

The observed and expected counts for the validation data were most similar for models 2 and 3 (Table 4). This comparison revealed that our predictive models could accurately predict the number injuries or deaths in future farm crash data from the same nine US states.

**Table 4:** Validation of the predictive models

Validation data years (Training data years)	Total validation data occupants (training data occupant)	Observed injuries or deaths in validation data (*N*)	Non-modifiable (Model 1)		Non + semi-modifiable (Model 2)		Non + semi + modifiable (Model 3)	
			Avg. pred. Prob.	Expected injuries/ deaths (*N*)	Avg. pred. Prob.	Expected injuries/ deaths (*N*)	Avg. pred. Prob.	Expected injuries/ deaths (*N*)
2008-‘10 (‘05-‘07)	7624 (7210)	1095	0.1407^b^	1073	0.1445^e^	1102	0.1437^h^	1095
2009-‘10 (‘05-‘08)	5216 (9618)	779	0.1383^c^	721^a^	0.1405^f^	733	0.1397^i^	729
2010 (‘05-‘09)	2615 (12,219)	372	0.1424^d^	372	0.1410^g^	369	0.1401^j^	366

*Abbreviations*: *Avg. pred. Prob.* Average predicted probability

^a^*p*-value = 0.0327 (Chi Sq = 4.56, df = 1), suggesting that expected injuries and deaths (*n* = 721) were significantly different than the observed (*n* = 779). All other expected to observed differences were non-significant

^b^AUC = 0.69 based on training data 2005–2007

^c^AUC = 0.70 based on training data 2005–2008

^d^AUC = 0.69 based on training data 2005–2009

^e^AUC = 0.75 based on training data 2005–2007

^f^AUC = 0.75 based on training data 2005–2008

^g^AUC = 0.74 based on training data 2005–2009

^h^AUC = 0.77 based on training data 2005–2007

^i^AUC = 0.77 based on training data 2005–2008

^j^AUC = 0.77 based on training data 2005–2009

Examining model fit as shown in Table 3, and validation in an external dataset (external to the training dataset) as shown in Table 4, are important pieces in addressing potential underfitting or overfitting of the predictive models. With an underfitted predictive model, within the training data, the expected model outcome counts will not accurately represent the observed counts due to bias. This may happen due to consideration of a limited number of predictors, or due to collapsing variables with multiple discrete categories into a few large categories that may not represent the finer categories. An adequate model fit will exhibit a close correspondence between the expected and observed counts for the training data (Table 3). On the other hand, one can include too many predictors (and interactions of those predictors) to achieve a nearly exact model fit, so that the expected model outcome counts from the training data nearly replicate the observed outcomes from the training data. When applied to external validation datasets, such overly complex models may not be able to accurately estimate the new observed outcome counts. These types of predictive models are referred to as overfitted or non-generalizable. In this example, the models accurately approximate the observed counts for both the training dataset (Table 3) and the validation dataset (Table 4). The propriety of the model fits is also represented by the AUCs.

Using predicted probabilities based on model 3, we constructed a hypothetical crash scenario to estimate the probability of injury or death for the involved individuals with specific demographic and driving characteristic profiles. We consider the following non-modifiable characteristics for the scenario: male driver of a farm vehicle in Iowa, aged 25–34 years, was involved in a single vehicle farm crash on a clear morning (6:00–11:59 am) during the growing season (June–August). Semi-modifiable characteristics: the manner of collision set to non-collision, the vehicle action set to heading straight, and the presence of a male passenger. Modifiable characteristics: Driver contributing circumstances set to none and occupant protection set to all occupants wearing seatbelts (Table 5). In this scenario, the risk of injury or death for the farm vehicle driver would be 16.7% and that for a male passenger would be 17.8%. If, however, the driver and passenger were not wearing seatbelts, and the driver disregarded the traffic regulations, their respective risks of injury or death would be 48.5% and 50.4% (Table 5). Suppose this scenario was altered to be a multivehicle crash in which the farm vehicle (with the same male driver and male passenger) was rear-ended by a non-farm vehicle (with a male driver and a male passenger). If all the individuals were wearing seat belts, and both the drivers were following traffic regulations, then the risk of injury or death in the crash would be 7.3% for the farm vehicle driver, 7.8% for the farm vehicle passenger, 28.7% for the non-farm vehicle driver, and 30.3% for the non-farm vehicle passenger. If these individuals were not wearing seatbelts and the drivers did not follow traffic regulations, then their respective risks would be 26.9, 28.4, 65.3, and 67% (Table 5).

**Table 5:** Predicted probabilities of injury or death for drivers and passengers in varying farm crash scenarios using the model 3 estimated coefficients from Table 1

The risk or injury or death in a farm crash in Iowa for a 25–34 year old, male, in growing season, clear weather, between 6:00–11:59 am, daylight, heading straight, passengers on board (for single vehicle crash, manner of collision = non collision; for multiple vehicle crash, manner of collision = rear end)				
Vehicle/ occupant type	Seat belt	Driver contributing circumstances	Risk of injury or death (%)	
			Single vehicle crash	Multiple vehicle crash
Farm vehicle driver	Yes	None	16.7%	7.3%
Farm vehicle passenger			17.8%	7.8%
Non-farm vehicle driver				28.7%
Non-farm vehicle passenger				30.3%
Farm vehicle driver	Yes	Disregarded traffic regulations	22.2%	10.1%
Farm vehicle passenger			23.6%	10.8%
Non-farm vehicle driver				36.4%
Non-farm vehicle passenger				38.2%
Farm vehicle driver	No	None	39.8%	20.6%
Farm vehicle passenger			41.6%	21.8%
Non-farm vehicle driver				57.0%
Non-farm vehicle passenger				58.8%
Farm vehicle driver	No	Disregarded traffic regulations	48.5%	26.9%
Farm vehicle passenger			50.4%	28.4%
Non-farm vehicle driver				65.3%
Non-farm vehicle passenger				67.0%

**Fig. 1:**
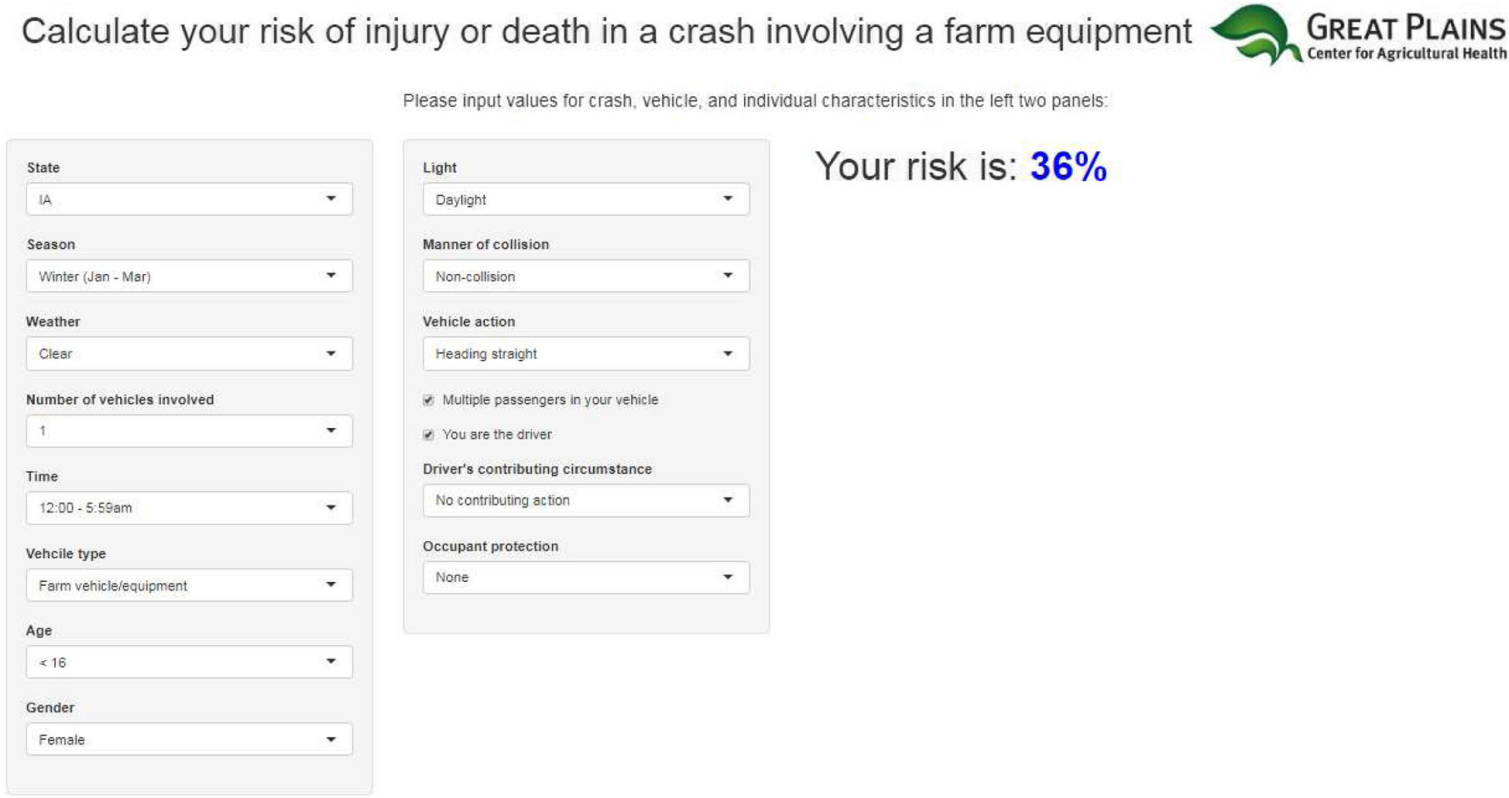
Screenshot of the online tool to calculate risk of injury or death in a farm crash

These risks can be easily estimated by deploying interactive tools that utilize model coefficients from the final fitted model. We developed a Microsoft Excel-based tool using the model 3 coefficients from 2005 to 2010 data that can be downloaded at https://drive.google.com/drive/folders/0B0B0TgPTSgJ8bjVZYjdGaF96UzQ. We also developed a similar internet-based tool using R-Shiny (Fig. 1); the online tool can be found at https://gpcah-farmcrash-predictive.shinyapps.io/predictiveapp/. These two tools are freely available and can be used by anyone to estimate an individual’s risk of injury in a farm crash. The risk estimates in Table 5 were produced using these tools.

## Discussion

In this manuscript, we demonstrate how the regression coefficients obtained from predictive analyses can be used to forecast the risk of a health outcome for individuals in specific farm crash scenarios. The predictive models allow us to compare the risks in different scenarios, helping us to appreciate the change in risk with varying circumstances as shown in Table 5. Specifically, the change in modifiable and semi-modifiable factors may allow us to understand the impact of intervening on these factors on the individual’s risk of injury or death.

In predictive analyses, the estimated model coefficients may be used to suggest that a certain variable is a strong or weak predictor of the outcome; however, the coefficient estimate is not used to characterize the effect of a variable on the outcome (which is the aim of causal modeling). This fundamentally differentiates predictive modeling from causal modeling, with the latter being ubiquitous in the public health literature. In predictive modeling, a coefficient affects the probability of the outcome for every individual differently based on all other individual or scenario-specific coefficients (Table 5).

Predictive modeling is used to ultimately answer the questions like “what is the probability (or risk) of an injury for an individual given a crash and the combination of all circumstantial factors?” It is noteworthy that the risks estimates presented in Table 5 do not entirely depend on driver behavior. They also depend on the total contribution of risk factors such as driver or passenger (semi-modifiable factor), farm or non-farm vehicle (non-modifiable factor), and single vehicle crash or multivehicle crash (non-modifiable factor). In addition, in this example, other characteristics were considered constant for all the vehicle occupants, but they may be changed easily (and will result in different risk estimates) to determine risks for other individuals in different scenarios.

The non-modifiable, semi-modifiable, and modifiable factors used to predict the injury or death in our example dataset have been previously identified as risk factors for a farm crash (Ranapurwala et al. 2016; Hughes and Rodgman 2000; Peek-Asa et al. 2007; Pinzke and Lundqvist 2004; Costello et al. 2003; Gerberich et al. 1996; Jaarsma and De Vries 2014), and injury (Hughes and Rodgman 2000; Marlenga et al. 2006; Gkritza et al. 2010; Maio et al. 1992; Stephan and Newstead 2014; Russo et al. 2014). However, these studies focused on reporting effect estimates (odds ratios or rate ratios) of association between individual risk factors and injury or death in a farm crash. Such models also assume that an individual’s outcomes do not depend on another individual’s outcomes (no interference assumption, also known as the stable unit treatment value assumption) (Schwartz et al. 2012). This is another differentiating feature, that while causal (or associational) modeling assumes (rather requires) no interference, predictive modeling embraces interference.

Using different combinations of predictors could allow one to estimate the predictive capability (or sensitivity) of the predictive models. For example, we identified realistic scenarios where the risk of injury given a farm crash was as low as 3% for some occupants, and as high as 93% for others, a difference of up to 90% of injury and death risk. This suggests good predictive capability of the model, since the predictive model covers most of the probability range between 0 and 1 (or 0 and 100%). Such a sensitive tool may be useful for policy makers to simulate potential effects of evidence-based interventions in well-defined populations and conduct cost-benefit analysis for widespread policy initiatives. For example, if an intervention is known to improve seat-belt compliance by 60% among a specific group of low compliant drivers, it could be simulated using our predictive model, which will allow us to estimate the number of overall injuries and deaths prevented. We can then estimate the cost of the prevented injuries and deaths and compare that against the cost of implementing the intervention.

Similar predictive models could be developed in other research areas to help physicians make more informed decisions about prescribing medications to their patients. For example, a risk prediction tool, like the one we developed, could inform physicians of their patient’s risk of suffering from an opioid use disorder in the future due to exposure to prescription opioid medication. Such information will help the physician to better titrate the patient’s pain medications. Eventually, such tools may not only aid physicians but also improve health outcomes for patients. Now, with the advancement of analytical approaches and superior computing power, and their integration in medicine, such approaches can be feasibly deployed and used.

Some best practices to develop and improve predictive models may involve the following, although this is not an exhaustive list. First, the overall data sample should be carefully selected so that it represents the relevant source population. Second, the training dataset should be large enough so as to observe all possible combinations of predictors. Third, the validation dataset should represent the same data generating mechanism as the training dataset, which can be accomplished through random selection of the observations for the validation dataset from the overall sample. Fourth, for quantitative variables, the most appropriate functional or categorical representation must be utilized. For example, age broken into 5-year categories may sometimes provide better prediction than 10-year age categories or age treated as a continuous variable. Fifth, when predictive outcomes exhibit secular time trends, appropriately accounting for such trends in the modeling structure may help improve the prediction. Sixth, when combining finer categorical variables into larger categories, it is prudent to combine finer categories that produce similar model coefficients and avoid combining categories that vary considerably in their model coefficients. The use of a model selection criterion, such as QIC, AIC, or BIC, can often facilitate the fourth, fifth and sixth objectives. Lastly, for a predictive model to be utilized in practice, there must be a temporal ordering between the predictors and the outcome; specifically, all predictors must be observed before the occurrence of the outcome.

These individual-level predictive models are extended further into more complex modeling frameworks like agent-based models that predict systems level changes. Historical and existing data sources can be used to develop predictive models, which can incorporate more data over time to improve the model’s predictive capabilities. Such iterative updating can be viewed as a type of “machine learning” (Wicks et al. 2016). Advances in computing have already taken predictive modeling to the next stage, via machine learning and precision medicine. Methods such as random forests, neural networks, and Q-learning utilize observational or experimental data, superimpose predictive modeling theory, and not only predict potential future outcomes, but also allow us to make evidence-based decisions to optimize the best possible outcomes in the future. However, some machine learning methods have been characterized as “black box” techniques that obfuscate rather than illuminate the dynamics of the underlying phenomenon. A clear advantage of the predictive modeling approach presented here is that it provides an interpretable and transparent characterization of these dynamics (Keil and Edwards 2018).

### Limitations

We encountered a number of limitations in our example dataset that commonly arise in predictive modeling regardless of the data source or substantive area of research. First, these data were collected from nine US states; hence, the results are not generalizable to other US states. Second, the crashes included in these data are those that were reported to the police. Some crashes may never be reported, misreported as non-farm crashes, or be excluded due to missing information. Similarly, mistakes may materialize in the reporting of injury severity by the responding police officers. Such exclusions and measurement errors may lead to misclassification of the predicted probabilities. Third, we developed the predictive models using commonly available variables form the nine states; however, there may be other unmeasured variables that may predict the outcome and hence may have an important role in determining the risk of injury for an individual. Generalizability, misclassification, and unmeasured predictors are three main limitations that may exist with most predictive models.

## Conclusion

Predictive analysis offers elegant tools that may help us understand the multidimensionality of the occurrence of different health outcomes and allow individual-level risk assessment. Algorithms and tools derived from such analyses could support policymakers, physicians, and environmental health practitioners in developing and implementing tailored prevention strategies to improve health outcomes for their patients or clients.
